# Printed Electronics as Prepared by Inkjet Printing

**DOI:** 10.3390/ma13030704

**Published:** 2020-02-04

**Authors:** Vimanyu Beedasy, Patrick J. Smith

**Affiliations:** Laboratory of Applied Inkjet Printing, University of Sheffield, Sheffield S1 4BJ, UK; vbeedasy1@sheffield.ac.uk

**Keywords:** inkjet printing, printed electronics, droplet behavior, ink, laser sintering, electrical conductivity, adhesion

## Abstract

Inkjet printing has been used to produce a range of printed electronic devices, such as solar panels, sensors, and transistors. This article discusses inkjet printing and its employment in the field of printed electronics. First, printing as a field is introduced before focusing on inkjet printing. The materials that can be employed as inks are then introduced, leading to an overview of wetting, which explains the influences that determine print morphology. The article considers how the printing parameters can affect device performance and how one can account for these influences. The article concludes with a discussion on adhesion. The aim is to illustrate that the factors chosen in the fabrication process, such as dot spacing and sintering conditions, will influence the performance of the device.

## 1. Introduction

Like many areas of human endeavor, the field of printed electronics is full of promise and challenge. The promise is a simple method of producing a wide range of electronic circuits and devices on large flexible substrates cheaply. The challenge is a continuing drive to improve performance, increase manufacturing speed, and identify new applications.

In this article, the reader is first introduced to the method of manufacture: printing. Several of the principal printing techniques will be discussed with an emphasis placed on inkjet printing, as that is a particular research interest of the authors. The term ‘printed electronics’ will be then defined along with a survey of the application areas, as well as an overview of the ongoing research in the field of printed electronics.

The focus of the article will then move onto an overview of the two principle materials groups used in printed electronics. The first group is the inks, and this section will elaborate on conductors, insulators, semi–conductors, and dielectrics. The second materials group highlights the various substrates that have been employed.

The discussion then moves onto the performance of the printed devices with the majority of the study being concerned with electrical performance, particularly conductivity. Adhesion is also addressed before some concluding remarks are delivered.

## 2. Printing

The process described as printing involves the controlled deposition of a material, either for decorative or functional purposes, onto a substrate in such a manner that a pre-defined pattern is produced. Other deposition processes, such as painting or spraying, have much in common, but printing is further defined due to the fact that the process can rapidly produce identical multiples of the original. (As a pleasant aside, the reader is referred to the career of Albrecht Dürer, who via the medium of printmaking gained a greater audience for his work, as well as a greater income.)

**Figure 1:**
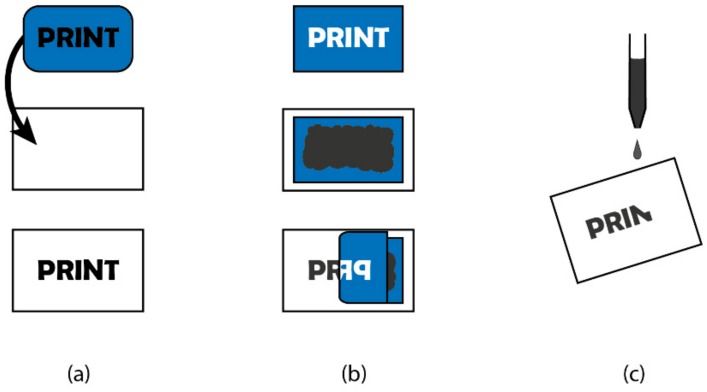
Illustration of the three printing techniques. (**a**) represents the positive contact printing similar to stamping, (**b**) represents the negative contact printing similar to screen printing, and (**c**) represents non-contact printing similar to inkjet printing, where ink is ejected from a nozzle.

There are three basic methods of printing, which can be described as positive contact printing, negative contact printing, and non-contact printing, as shown in Figure 1. The first two methods are described as contact printing since the substrate is touched by the print master. In positive contact printing (commonly called relief printing), a master of the desired image or feature is produced. The feature is, typically, raised above the surface of the rest of the master, and is the region of the master that holds the ink. Although the production of this master takes time, the user is compensated by the subsequently rapid manufacture of the prints. Once obtained the master is inked and then pressed onto a substrate. Examples of positive contact printing include printing presses and woodcuts. It should be noted that with these and other examples, the print that is produced is a mirror image of the master.

For negative contact printing, a master is still produced, but the areas that are printed are either recessed into the surface, or are in the form of voids in the master. Here, the surface of the desired feature is lower than the rest of the surface of the master. As examples, gravure printing involves the use of a patterned drum, with the recesses that define the pattern being filled with ink [1]. Screen printing is another example, in which a mesh is placed over the substrate. The mesh has been previously patterned with a negative of the desired print. Ink is then placed on top of the mesh to one side and pushed onto the substrate by a wiper travelling to the other side.

Non-contact printing is named as such due to the fact that a master does not make contact with the substrate. Examples of non-contact printing involve inkjet printing (IJP) and aerosol jet printing (AJP). Many, if not all, of the non-contact printing techniques do not involve the use of a master, which affords them great versatility in terms of incremental, or radical, changes to a print. Both the IJP and AJP technologies have been exploited in various industries, such as textile, graphics, and medicine, but the field of printed electronics is where they have been of greater use [2,3]. A detailed comparison of both technologies done by Seifert et al. [4] for printed electronics applications highlighted the pros and cons of IJP and AJP. While IJP generally produces line widths as small as 20 µm [5,6] on hydrophobic surfaces, AJP can produce even thinner lines down to 10 µm [7]. Moreover, AJP can print higher viscosity inks of up to 1000 cP as compared to IJP, which can only print viscous inks between 5–20 cP [8], enabling a wider range of functional materials to be used in AJP. The advantages of AJP make the technology particularly suitable for printed electronics applications [9,10] yet it is much less established than IJP and there are significantly less research findings covering AJP, mainly because the technology requires a much higher start-up cost.

In terms of advantages and disadvantages, the contact printing methods generally have faster throughput but higher start-up costs due to the need to produce a physical master. Non-contact printing techniques have significant advantages in terms of small batch runs due to the lack of a master; they can also switch rapidly from one pattern to another, as well as being able to deposit more than one color in a single printing step. Non-contact printing techniques also have the advantage that potential sources of contamination are reduced or removed entirely.

## 3. Inkjet Printing

The deposition technique known as inkjet printing builds up images and structures in a droplet-by-droplet fashion. As a rule, there is minimal size variation between the produced droplets, unless the user makes a change to the jetting parameters or to the ink. This high degree of reproducibility in droplet production allows a user to treat the droplets as building blocks.

There are two families of inkjet printers: continuous inkjet and drop-on-demand inkjet. In continuous inkjet (CIJ), a constant stream of droplets is generated, passed between charging plates, acquiring an electrical charge as a consequence, then directed towards the substrate by an electric field if required. Since droplet production is continuous, there is a need to deal with unused droplets, which is met by capturing undirected droplets in a gutter; these captives are then recirculated back into the reservoir.

Drop-on-demand (DOD) inkjet is named as such since droplets are only produced when required, meaning that energy is more efficiently used and that the potential contamination due to the recycling of the unused droplets in CIJ is avoided. However, as there are times when the printhead is idle, clogging of the nozzle is possible due to solvent evaporation.

The method of generating the droplets in DOD leads to a division into two types. In thermal DOD, droplets are generated when a resistor in the ink chamber heats up vaporizing neighboring liquid and causing a bubble to form. In piezoelectric DOD, it is a pressure pulse formed in the ink chamber by the action of a piezoelectric actuator that ejects a droplet. The majority of industrial and research DOD printheads are of the piezo DOD type. Control over the velocity and the volume of an ejected droplet from a piezo DOD printhead can be achieved by varying the actuating voltage; as voltage increases, velocity and volume increase. However, as a rule droplet size is mostly determined by the diameter of the print nozzle, and if a user requires smaller, or larger droplets they can change to using a printhead with a corresponding size. Other parameters such as the waveform used for the droplet formation process also affect the volume and velocity of the droplet. Table 1 below classified the current inkjet printing systems based on their uses in either industry or in laboratory-based situations. The printheads that are manufactured for industrial applications are often combined with other in-house equipment from manufacturer’s to supplement their production flow. The other inkjet printing systems are the preferred choice for laboratory research as they are an all–in–one package with the printhead integrated in the print platform, together with the provided software. They have a lower footprint and generally consist of a single printhead, but can easily be scaled up for a multiple-printhead array.

## 4. Printed Electronics

Printed electronics can be defined as a manufacturing method of using novel materials, such as functional inks, to print onto a variety of substrates, leading to the fabrication of electronic devices. The production of printed electronics has been around for decades. In 1903, Albert Hanson, a German scientist, filed the first patent on printed wires in England and invented the circuit board for telephone systems, which consisted of conductive pieces of foil attached to wires and bonded to a flat sheet of paraffin paper [11,12]. The construction resembled modern printed circuit boards (PCBs), despite being “unrefined” by todays’ standards. From 1903 onwards, the production of PCBs was done via the use of stencils and electrically conductive inks, later produced via etching copper foils in 1943, and reinforced by soldering copper wires in 1961. In 1949, the U.S. Patent US2474988A, “Method of Manufacturing Electrical Network Circuits,” described how colloidal suspensions of tin are used to stamp conductive traces, and colloidal graphite coated by gelatin is printed to make resistors, to produce electronic circuits. As mentioned earlier, these methods are wasteful and require a high investment cost. The ongoing development of inkjet printing technology allows manufacturers and scientists to bypass these expensive traditional manufacturing methods, and as such, the field of printed electronics is benefitting from an increasing amount of resources and research [13,14,15]. There is some highly cited literature on printed electronics, such as works by Chen et al. [16] discussing the fabrication of conductive tracks with inkjet printing technology, Kamyshny et al. [17] documenting the use of metal–based inkjet inks for printed electronics, Huang et al. [18] reviewing the materials, processes and application of flexible and stretchable electronics, Khan et al. [19] studying the technologies for printing electronics over larger flexible substrates, and Gao et al. [20] focusing on wearable electronics.

The influx of printed electronics has mainly been influenced by advances in material technology where functional inks have been made more reliable for use by inkjet printing and other printing methods. Functional inks consist of an electrically relevant component that has either a semiconducting (organic and inorganic polymers), conducting (metallic particles), dielectric (ceramic–filled organic polymers), or insulating property and a carrier solvent.

An idea of the extent of printed electronic devices that can be produced can be found in organic LED displays [21,22,23], sensors [24,25,26] (glucose, RFIDs, pressure), smart textiles [27], thin-film transistors [28,29], thin-film batteries [30], intelligent packaging [31], and even photovoltaic cells [32,33,34]. The use of inkjet printing for the fabrication of these devices is due to it being a relatively fast technique with excellent resolutions, while minimizing the amount of waste produced. Inkjet can also be scaled up to be a roll–to–roll manufacturing process, although a compromise must be made for the post-processing step that requires either a long lead time or an elevated temperature. Other benefits of printed electronics include the ease of integrating into a manufacturing process, as the user does not require expensive pieces of equipment.

However, there are still challenges prevailing in the printed electronics field. There is still a significant amount of research into developing the most conductive ink and material for printed electronics applications in a cost-effective manner. This development process involves significant trial-and-error testing, which ultimately increases the cost of implementation.

## 5. Inks

The success of inkjet printing in the field of printed electronics is attributed to functional, printable inks. These can be categorized based on their constituents’ dimensional nanostructured materials: *0-D* (zero-dimensional) materials are those which conform to the nanoscale and are typically less than 100 nm (e.g., nanoparticles); *1-D* (one-dimensional) materials are those outside the nanoscale (e.g., nanowires, nanotubes); and *2-D* (two-dimensional) materials exhibit plate-like shapes (e.g., graphene) [35].

### 5.1. Metallic Inks

Some of the main materials to be considered when forming conductive tracks, which are essential in printed electronics, are metals. Metallic nanoparticle inks (zero-dimensional materials) are available commercially and are based on a feedstock of nanoparticulate metal. The main advantages of nanoparticle ink are its stability, leading to a good shelf life; high loading of up to 40 wt.%, meaning more metal can be deposited per pass of the printhead; and lower contact resistance upon sintering [36], [37]. Metallic nanowires (one-dimensional materials) exhibit similar properties in terms of shelf life, although there are fewer papers describing the use of metal nanowires compared to metal nanoparticles in inkjet printing. This is due to the high aspect ratio of the wires that cause the jetting nozzles to clog, and due to the lower volume fraction of nanowires in an ink, the printed pattern requires multiple passes to produce an electrically conducting part. Metal nanowires are preferred to nanoparticles owing to their higher mechanical ductility [38,39] and are used to produce antennas [40] and wearable electronics [41].

Metal solutions, often called metal organic decomposition or metal−organic decomposition (MOD) inks, involve a metal salt dissolved into a suitable solvent. MOD inks deliver higher conductivities, and since they are solutions, result in reduced nozzle clogging [42].

After deposition, both types of ink can be thermally converted to the conductive metal using temperatures of 200 °C or lower. Much research has been performed focusing on lowering the processing temperature with both types of ink able to be converted at room temperature, allowing a wide range of heat sensitive substrates to be used [43].

A number of strategies can be employed to increase conductivity, with one of the simplest being to print additional layers, which also reduces variability in the values of conductance. Higher processing temperatures can also help, as can decreasing the dot spacing parameter of the inkjet printer, which leads to more functional materials per unit area.

#### Choice of Metals

In terms of which metal to use, the decision is primarily dictated by cost, performance, and ease of handling. Silver, although the most conductive of metals, is expensive, whereas copper, which is much more affordable, often requires a controlled atmosphere in order to prevent the formation of copper oxide.

In terms of bulk resistivity, silver has the lowest, 1.59 × 10^−8^ Ω·m, copper has the next lowest 1.72 × 10^−8^ Ω·m, then gold 2.44 × 10^−8^ Ω·m. Although, all of these three metals have excellent values of conductivity, once price is accounted for in the attraction of each of the changes, gold, as expected, costs about a thousand dollars per ounce, silver is about twenty dollars an ounce, whereas copper is only about twenty cents an ounce. Aluminum (2.82 × 10^−8^ Ω.m and about ten cents per ounce) and nickel (6.99 × 10^−8^ Ω·m, about fifty cents an ounce) have also been considered [43,44].

### 5.2. Non–Metallic Inks

The production of conductive patterns is not limited to the use of functional inks based on a metallic component. In fact, there are alternative polymer–based inks such as poly(3,4–ethylenedioxythiophene) doped with polystyrene sulfonate (PEDOT:PSS). An optimized formulation of PEDOT:PSS ink has many advantages over metal-based inks given its optical transparency (~88%), chemical stability, and structural elasticity (~1.2 GPa). After sufficient layers have been printed, the PEDOT:PSS pattern is comparable and consistent in terms of electrical performance and has been used in solar cells [45] and energy storage devices [46]. More importantly, PEDOT:PSS has proven to be biocompatible and has been used for lab-on-a-chip [47] and organ-on-a-chip [48] applications, as well as biocompatible stretchable devices [49].

Graphene, an allotrope of carbon and a non–metallic two-dimensional ink, has recently been in the spotlight for its promising properties: at only one atom thick (around 0.34 nm), it exhibits mechanical properties much stronger than either steel or diamond when a similar dimension is compared. Graphene has a tensile strength of over 1 TPa and yet is incredibly light at just 0.77 mg/m^2^. Being a 2D material, it is available in the form of sheets, which are flexible and can stretch up to 20% of its initial size elastically. The graphene sheets have excellent electrical properties and are perfect thermal conductors [50,51]. The production of graphene can simply be done via mechanical exfoliation, during which a piece of graphite (stacked layers of graphene) is repeatedly exfoliated using tape and transferred to a substrate. Other methods such as chemical vapor deposition (CVD), liquid phase exfoliation, electrochemical exfoliation, chemical reduction of graphene oxide, and bottom–up synthesis have proven successful, but are more elaborate steps. The reader is referred to the paper by Coroş et al. [52] for a more in-depth study of the different methods of synthesizing graphene. Graphene has been integrated in thin-film transistors in the form of electrodes [53,54], sensors, and energy storage devices [55,56]; and more importantly it has been combined with PEDOT:PSS to produce biocompatible, skin-comfortable temperature sensors [57,58].

The fabrication of printed electronics is not limited to conductive inks. In fact, the use of insulators plays a vital role in maintaining optimum device performance. For example, in thin-film transistor (TFT) devices, where two electrodes are separated by a few µm, it is crucial for these electrodes to remain separated, and this is achieved by printing a layer of insulating polymer on top. It is important that the polymer does not react with the electrodes or the substrate, or any subsequent layers printed on top. In some cases, the insulating material printed is a monomer, which is then converted to a polymer via heat treatment.

Insulators can act as barriers between stacked or adjacent layers and can also act as encapsulating layers. UV curable resins (e.g., SU–8, EMD6415, aluminum oxide–resin composite, epoxy–based resins) [27,59,60,61] have been used for devices such as metal–insulator–metal capacitors and for smart textiles. Polydimethylsiloxane (PDMS) is another widely used silicon-based organic polymer that is employed for its insulating properties as well as its hydrophobicity after cross-linking [62].

There are some exceptional cases where the insulating material can be a variant of a polymer, or simply another material. In 2015, Jang et al. [63] inkjet-printed zirconium dioxide, a metal oxide, as an insulator material in a TFT device. Polyaniline is an exceptional polymer, highly researched, which exhibits opposing properties upon protonation [64,65]. It exhibits conducting behavior when doped with electrons, named ‘polyaniline–e’, and when oxidized, it changes from the emeraldine form (polyaniline–e) to the non–conductive pernigraniline form, named ‘polyaniline–p’. This intriguing behavior has been exploited in the fabrication of printed electronic devices [66].

In capacitor and transistor printed electronics, dielectrics are used to maintain an electrostatic field while minimizing the energy loss in the form of heat. In practice, most dielectric materials are solids (e.g., ceramic, mica, metal oxides), but in printed electronics these dielectric materials are in the form of polymers. Poly(4–vinylphenol) (PVP) is commonly used for the fabrication of organic TFT devices due to its solubility in aqueous suspensions and its low temperature processing requirements. Klauk et al. [29] reported better electrical properties using PVP gate dielectric layers than SiO_2_ gate dielectric layers [67]. However, PVP is not stable at ambient conditions owing to its poor resistance to humidity, thus it requires stabilizers such as poly(melamine-coformaldehyde) methylated (PMF), working as a cross-linking agent, in propylene-glycol-monomethyl-ether-acetate (PGMEA) [68].

Other dielectric materials used in inkjet printing include TIPS-Pentacene, which is readily available from numerous manufacturers. High purity TIPS-Pentacene is used in the fabrication of organic field-effect transistors (OFETs) using inkjet printing methods, owing to its excellent solubility in a range of common organic solvents and its stability at normal conditions.

## 6. Substrates

Once the user is satisfied with the printing parameters for the desired ink, the next step is to define the resolution of the print on the substrate of choice. At a laboratory scale, it is common to print on glass slides as they are non-porous, non-reactive substrates and offer a relatively smooth adhesive surface for the inks to be printed on. Glass is a relatively inexpensive option and its obvious transparency allows the printed features to be clearly observed from both the top and the bottom. In the field of printed electronics, inks often undergo some level of post-processing such as a heat treatment or chemical washing, hence the substrate needs to be compatible with the process. Glass slides, being made from silica, can withstand temperatures around 513 °C until they begin to strain, which is well above the sintering temperatures of most functional inkjet inks (200–300 °C). When quantifying the electrical and topological properties of functional metal nanoparticle inks, it is important for the substrate not to influence those properties.

Contrary to glass, paper is a porous and rough substrate of choice, yet is often preferred for being flexible, inexpensive, and recyclable. The applications range from flexible sensors [69], security printing [70], microfluidics [71], and even biomedical [72] applications. Despite the porosity of paper-based substrates, the addition of coatings has drastically improved the printing resolution as well as the functioning behavior of the ink. For example, inkjet paper [73] has a thin coating of a resin which “traps” the nanoparticles of the ink onto the surface while absorbing the fluid content through the paper, resulting in a narrow resolution print and a glossy or matt finish. However, the sintering process is highly limited; as paper is made from cellulose, it thermally decomposes at temperatures above 100 °C, and ignites at 233 °C.

Instead, in printed electronics, the more reliable and commonly used flexible substrates tend to be polymeric by nature. Polyimide (PI) substrates have been in circulation for several decades and are preferred for their wide thermal stability (from −269 °C up to 400 °C), flexibility, and robust mechanical properties under harsh conditions. DuPont™ Kapton^®^ foil is the most commonly used PI substrate, which can be formed into thin films ranging from 25 µm to a few millimeters, and is used in various fields of electronics and even in the international space station [74]. However, PI is almost opaque by nature and is not desirable for applications which require transparency. Other polymers such as semi-crystalline polyethylene terephthalate (PET) and polyethylene naphthalate (PEN) are at the forefront in the development of flexible electronics. They are available in a range of film thicknesses. Even though the thermal properties of PET and PEN do not match that of PI (melting points of PET and PEN are 255 °C and 270 °C, respectively), their chemical resistance to solvents and intrinsic clarity make them suitable for applications that require transmission of light and electrical conduction [75].

Polydimethylsiloxane (PDMS) is another silicone elastomer substrate that is used for stretchable electronics given its elastic properties [76]. It is optically clear and generally inert, and non-toxic, as such it has been used for biocompatible applications [77]. Despite being highly hydrophobic in nature (contact angle 90°–120°), it can be reconfigured through surface treatments using other polymers to improve its surface wettability and adhesion to metallic inks [78].

## 7. Wetting

The substrate is an active partner in defining the quality of a print. In particular, it is the energy of the substrate that determines whether an end user is satisfied with their print and whether the printed device will function as intended. “Wetting” is a phenomenon which defines how a liquid spreads across a surface. A poorly wetting ink does not spread and breaks up into beads, as a consequence of the low energy it experiences with the surface. Conversely, an ink may wet the substrate too well, which can lead to a phenomenon called “coffee staining”. In both cases, the key issue is the distribution of the functional material carried by the ink. Where the substrate has a low surface energy, the functional material takes the form of randomly positioned clumps and agglomerates. Where the substrate has a high surface energy, most of the deposited functional material flows to the boundary of the drying print.

**Figure 2:**
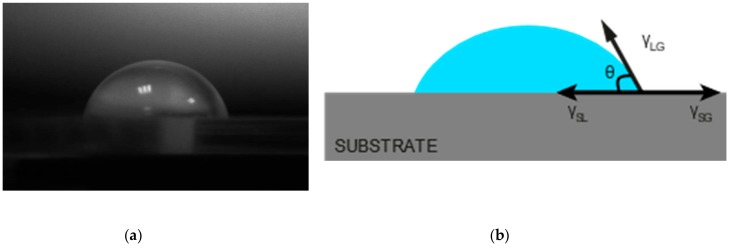
(**a**) Picture of a sessile drop in equilibrium on a polymer substrate. (**b**) Representation of the interfacial forces acting on a droplet on a substrate. The contact angle, *θ*, is less than 90°, indicating a partial wetting of the substrate.

The surface energy experienced by a liquid can be simply observed by looking at the angle formed by a droplet of the liquid with the substrate of interest, as shown in Figure 2. The interfacial tensions, γ_xy_, determine the shape of the droplet from the three interfaces liquid-gas (LG), solid-gas (SG), and solid-liquid (SL).

The equilibrium contact angle, *θ*, can then be calculated using these interfacial tensions into the static Young’s equation [79]:

(1) $$\cos\;\left(\theta \right)=\;\frac{\gamma_{SG}\;-\gamma_{SL}\;}{\gamma_{LG}}$$

**Figure 3:**
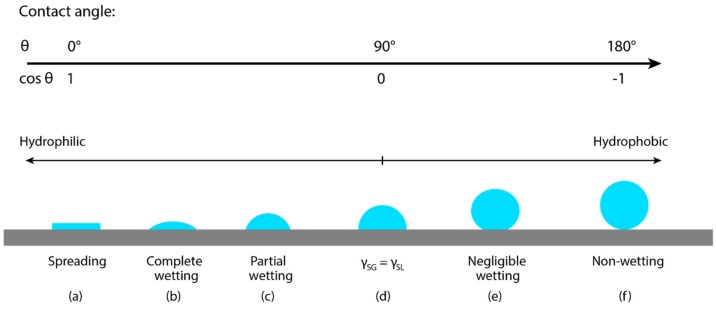
Representation of the various scenarios which can occur when a droplet is deposited onto a dry solid surface, illustrating the possible wetting states.

The wetting state of the fluid can be determined once the interfacial tensions are known. Based on Young’s equation above, if γ_SG_ < γ_SL_ + γ_LG,_ then a droplet will spread onto the surface, leading to partial wetting (Figure 3c). On the other end, if γ_SG_ > γ_SL_ + γ_LG,_ then a droplet will form a spherical cap resting on the surface, leading to negligible wetting (Figure 3e), similar to a hydrophobic surface. Another scenario would be when γ_SG_ = γ_SL_ + γ_LG_, leading to a contact angle of zero, resulting in complete wetting of the solid substrate as the thermodynamic system will be in complete equilibrium (Figure 3a) similar to a hydrophilic surface. Assuming the volume of the ejected droplet is conserved, the degree of droplet spreading can be calculated using:

(2) $$\beta_{eqm}=\frac{d}{d_{eqm}}={\left(\frac{8}{\tan\left(\frac{\theta }{2}\right)\left(3+\tan^{2}\left(\frac{\theta }{2}\right)\right)}\right)}^{\frac{1}{3}}$$

where *β_eqm_* is the ratio of the initial droplet diameter, *d*, to the diameter of the droplet on the substrate at the equilibrium contact angle, *d_eqm_*.

**Figure 4:**
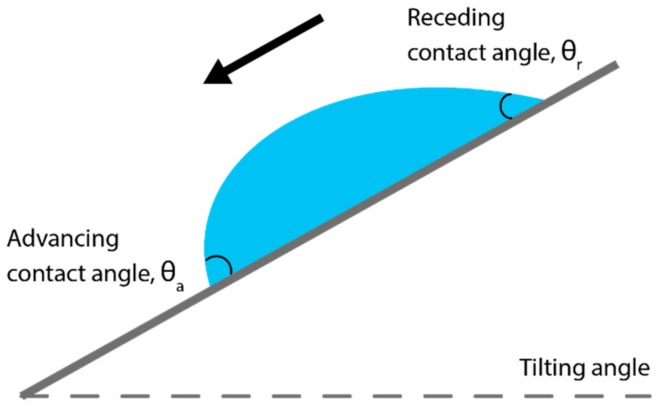
Illustration of the different angles formed by a droplet on a substrate.

There are three angles formed by a sessile droplet: advancing, equilibrium, and receding, as shown in Figure 4 below.

The advancing angle, θ_a_, is formed by a droplet that is growing, which means that more liquid is being added to the initial droplet, causing the droplet to advance over the substrate. The advancing angle can also be formed at the front of the droplet if the substrate it is sitting on is tilted, or there is a depression.

Conversely, the receding angle, θ_r_, is formed by a droplet that is losing mass, such as due to evaporation, causing the contact line of the droplet to retreat. Similarly, θ_r_ is also formed at the back of a droplet on a tilted substrate. The contact line is the boundary of the droplet, or liquid feature, and is the interface between the liquid of the droplet, the gas, and vapor of the local atmosphere and the solid of the substrate.

The equilibrium angle is the angle formed by the droplet when it has stopped growing/shrinking and is not moving, which is the angle most people refer to when they talk about an ink’s contact angle.

**Figure 5:**
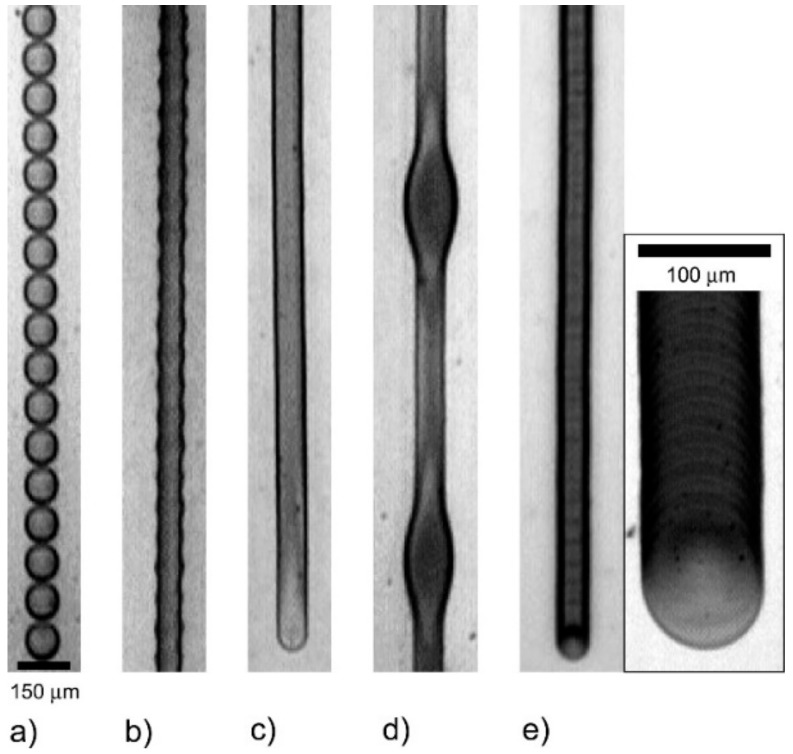
(**a**–**e**) Illustration showing the relationship between dot spacing and print morphology. Reprinted (adapted) with permission from [80]. Copyright (2019) American Chemical Society.

In an inkjet-printed system, the amount of ink deposited per unit area can be determined by the dot spacing, which is defined as the distance between the centers of two droplets. Another term for dot spacing is dots per inch (often abbreviated to ‘dpi’). Soltman and Subramanian [80] investigated the effect of varying the droplet spacing on the print morphology and their findings can be summarized using Figure 5 below. If the drops are printed too far apart at a distance greater than their diameter, it results unsurprisingly in the formation of isolated drops that dry separately (Figure 5a). Decreasing the dot spacing causes the drops to merge with the formation of “scalloped” lines (Figure 5b); further decreasing the drop spacing leads to uniform lines with straight edges (Figure 5c). Past this point, if the droplet spacing is decreased even more, there is an increase in the overlap between droplets and discrete bulging along the line is observed, particularly at the beginning (Figure 5d). Finally, if the substrate temperature is increased such that the evaporation time of a droplet is shorter than the drop jetting period (or simply that the time between each droplet ejection event is longer than the drying time), this leads to each drop drying individually and results in a “stacked coin” morphology (Figure 5e). In this regime, drop spacing has no effect on the width of the printed lines as each drop is dried prior to the deposition of the next drop.

As with every problem, in the case of over-wetting or under-wetting of a substrate, scientists have demonstrated methods of tailoring the surface energy of the substrate via plasma or UV/ozone treatment. The advantages of using a surface treatment prior to printing greatly improves the resolution of the structures by facilitating the self-alignment of ink, but they add an additional process step and often require expensive equipment and facilities. Nguyen et al. [81] demonstrated high wettability contrast (100° water contact angle difference) of inkjet-printed PEDOT:PSS on PET substrates using a combination of different surface treatment methods. Park et al. [82] investigated surface treatments using O_2_ and plasma on PI films and printed silver nanoparticle inks, resulting in a control of the drop size variation between 38 µm to 70 µm. Similar studies were performed by Lee et al. [83] using UV/O_3_ surface treatments on fluorocarbon films, resulting in a controlled deposition of silver nanoparticle ink on the hydrophobic surface. Ta et al. [84,85] investigated the use of a nanosecond laser to treat the surface prior to printing, and as such created a surface with varying roughness and wettability, which is useful in sensors. From a printed electronics point of view, the control of the wettability of a surface is crucial to optimize the print resolution and hence prevent the formation of defects which would result in a waste of resources.

## 8. Processing

Once the functional inks have been printed, they usually require some level of heat treatment to improve the characteristics of the final product. These can either be the electrical properties, mechanical structure, or surface finish, or a combination. For inks serving a conductive purpose, the sintering step is crucial to remove the non-metallic component which acts as barrier to the flow of electrons throughout the printed feature. In the context of printed electronics, the term “sintering” refers to the removal of surfactants that prevent agglomeration of particles in the ink as well as particle growth and grain-boundary relocation. Nanoparticles (NPs) have a much lower melting point than the bulk metal given the surface area exposed to the heat is considerably larger. This is referred to as the thermodynamic size effect [86]. For example, gold nanoparticles with diameters less than 5 nm are predicted to melt well below 300 °C, which is considerably lower than the 1063 °C required to melt bulk gold [87]. Allen et al. [88] later showed that this reduction in the melting temperature is also valid for other metals including tin, lead, and bismuth. It was also found that plates, instead of spheres, do not show a reduced melting temperature. This suggests the dependence of melting on size in particles is related to the internal hydrostatic pressure caused by the surface stress and by the large surface curvature of the particles, but not by the planar surfaces of platelets [89].

**Figure 6:**
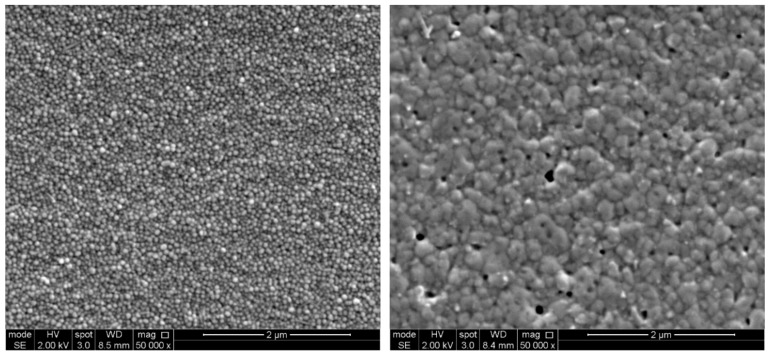
SEM of an inkjet-printed silver nanoparticle ink. The micrograph to the left represents the ink as printed, and the one to the right represents the ink after thermal sintering at 200 °C for 60 min.

The most commonly used sintering technique is thermal sintering as it is often the most reliable and readily available. This involves placing the printed feature in a convection oven or on a hotplate for a set amount of time at a certain temperature depending on the ink. For example, a commonly used silver nanoparticle dispersion ink requires a temperature of 150–200 °C for 60 min, according to the supplier. During this period, the first stage removes the organic solvent and dispersant from the printed ink. The dispersant prevents the nanoparticles from agglomerating, which is necessary for the ink to successfully print without clogging. Hence, during the first stage until the dispersant is completely removed, the nanoparticles remain discrete and only start to coalesce as the heating continues. During this stage, grain size increases until it reaches the final stage where the deposited metal is continuous. At the final stage, the grains have made sufficient contact with each other to ensure continuity in the printed structure as well as sufficient percolation pathways for electrons. However, the final structure is rarely 100% dense, as there will be some porosity due to the present of contaminants or surface imperfections, as shown in Figure 6.

Alternative sintering methods include the use of light in the form of radiation. An example of which is a LASER beam used to irradiate the printed feature at selective locations. Poulikakos et al. [90,91,92] used a continuous laser as part of an experiment to raster-scan selective regions on their printed feature to convert the irradiated area into conductive counterparts without damaging the underlying substrate. This method achieved conductivities of up to 25% of bulk gold and line widths of 8 µm were reported [93,94]. However, the completed product required an additional post-sintering step to wash off the unsintered material and proved to be relatively slow since the scanning process was done at translation speed of 0.2 mms^−1^ in order to obtain the highest conductivities [92].

Pulsed laser sintering, a method which used a laser diode pulsed at very short intervals (from picoseconds to milliseconds), has been used for a variety of purposes in inkjet printing. Contrary to the functioning of a continuous laser which has a stable power output, a pulsed laser is characterized by pulses of energy generated at specified frequencies. In the early 2000s, pulsed lasers were used to tailor the substrate and/or the printed pattern though a process called ablation, where the laser is pulsed at high frequencies to vaporize the materials [84,95,96]. With advances in the semiconductor material industry, pulsed laser diodes have become more reliable and scientists have better control over the pulsing parameters. Pulsed lasers, when combined with an inkjet printing system, have led to a direct fabrication method. When applied to the printed electronics field, scientists have produced conductive microstructures by pulsed laser sintering of metallic nanoparticles [90,91,92].

In the case of gold nanoparticle inks, the laser sintering process can be done at atmospheric conditions as the gold nanoparticles are stable and do not oxidize [97]. Silver nanoparticles are prone to oxidation, but the silver oxide formation does not inhibit the electrical properties of the printed silver pattern as silver oxide is conductive [98]. Copper nanoparticles; however, are very reactive at atmospheric conditions, especially on a nanometer scale, hence the formulation and sintering of the copper nanoink must be done carefully [99]. The sintering of copper has traditionally, and reliably, been done in a reduced atmosphere (e.g., nitrogen) with the use of a hotplate or an oven at elevated temperatures of 200 °C or above for at least an hour. Without the use of a reduced atmosphere, the lengthy sintering process would allow for copper oxide to form, resulting in a highly resistive sample.

By using pulsed lasers, the energy supplied in a pulse can be tailored to match the energy required for the dispersant in the copper nanoink to sublime and cause the formation of conductive necks in the printed sample. The sintering time is reduced drastically to picoseconds or nanoseconds, depending on the pulse width of the laser, in comparison to the conventional thermal sintering process and allows for selective locations on a sample to be irradiated.

Intense pulsed light (IPL) sintering is another method that uses radiation to produce conductive features after printing. The technique uses short pulses of intense light with a broad spectrum ranging from UV to IR, to increase the local temperature of the sample in milliseconds. Contrary to laser sintering, the converted area is relatively larger and the sintering process can be done without the need to raster the IPL beam. Mitra et al. [100] demonstrated the use of silver nanoparticle ink printed on PET foil and sintered using a flash lamp from Novacentrix, while Ryu et al. [101] presented a method using reactive sintering of copper nanoparticle ink using IPL to produce conductive copper patterns at ambient conditions on flexible polyimide film. A similar study detailing the use of copper nanoparticle ink printed on polymer substrates such as polyimide, polypropylene, and polyethylene films and sintered using IPL was reported by Kim et al. [102]. The bulk resistivities of copper and silver reported in these studies are comparable to those measured when the printed films were sintered using an oven at optimum conditions [103].

The advantage of photonic sintering over conventional thermal sintering is the reduction in the total amount of time needed for post-processing. More importantly, both LASER and IPL sintering have proven to limit damage to the substrate upon which the ink is printed, hence thermal-sensitive substrates such as polyethylene terephthalate (PET) and polypropylene (PP) films can be printed onto.

Microwave sintering is another method used to sinter inkjet-printed inks on a variety of substrate, but a thorough understanding of the process is yet to be concluded. Theoretically, the process can be explained by the Maxwell–Wagner effect [104] where the conductive particle interaction with microwave radiation results in the accumulation of charge at the material interface, electric conduction and eddy current. There have been numerous studies and models to gain a better understanding of the process [105], and some experiments have been proven to successfully produce conductive features on polymeric substrates [106,107]. The conductivity values obtained through microwave sintering are comparable to other sintering methods mentioned earlier.

The aim for a reduction in time and energy, and consequently process steps, has led to additional resources being invested in chemical sintering recently. With careful formulation of inkjet inks, the requirement for elevated temperatures as a post-printing finishing stage can be eliminated and instead replaced with a chemical immersion, which provokes nanoparticles to coalesce [108], or to moderately elevated temperatures ranging from 60–120 °C for 20 min [109]. Silver nanowires have recently been used to produce good performance flexible electrodes sintered under room temperature via different electrolyte solutions [110] on polycarbonate substrates. Recent development in ink formulation has resulted in the production of a self-sintering silver ink from Mitsubishi Paper Mill, whereby the conductivity of the ink spontaneously emerges as soon as the printed solution has dried [111] on a suitable substrate, with a quarter of the bulk conductivity of silver reported.

## 9. Device Performance

Colored inks, those which are used in a typical desktop inkjet printer in a household or an office, only require a visual analysis to determine whether the print is considered “successful” or not. However, in the case of functional inks, this visual inspection is only the first step. In the field of printed electronics, there are several methods of quantifying a completed print, each of which vary depending on the material printed. For conductive inks, the first stage is determining whether the sintering process is successful by inspecting the sample for surface deformation and cracks. Usually, the inkjet-printed sample contains particles on a nano (10^−9^) or micro (10^−6^) scale which can only be seen using an optical or electron microscope. If cracks are not present, the sample is then subjected to a series of performance tests including electrical resistivity, adhesion, and mechanical deformation. These performance tests are often destructive in nature.

A resistivity test is an absolute way of determining the true performance of the functional conductive ink printed. By definition, the electrical resistivity of a material is an intrinsic physical property which denotes its resistance to the flow of electricity and is independent of the shape of size of the sample. Resistivity, *ρ*, is measured in ohm·meters (Ω·m), and is expressed mathematically as:

(3) $$\rho =\frac{A}{L}\cdot R$$

where:

*ρ* = volume resistivity, *Ω·m*
*A* = cross–sectional area, *m^2^*
*L* = length, *m*
*R* = resistance, *Ω*

The resistance of a material is a measure of the degree of opposition to the flow of electric current through the material and is measured in *ohms*. Resistance measurements for conductors will vary according to the length, width, and thickness of the sample. As such, the preferred way of comparing the electrical characteristics of a printed sample is through the resistivity.

A material which has a low resistivity (e.g., copper wires) implies that electricity can flow easily, whereas one which has a high resistivity (e.g., glass) implies electricity does not flow well. Electrical conductivity is defined as the inverse of resistivity.

There are two devices which are used for measuring the conductivity of a sample: a two-point probe (i.e., an ohmmeter) and a four-point probe (i.e., a kelvin probe). The ohmmeter is accurate enough for samples where the measurement value is not close to the resolution of the meter. For example, a standard laboratory ohmmeter which may have a resolution of 0.1 ohms is not desirable for measuring a sample of 0.5 ohms. There is usually some lead resistance in the wires and some contact resistance between the probes and the sample, and the equipment itself. These additional resistances increase the resistance measurement of the sample leading to the inclusion of a systematic error.

**Figure 7:**
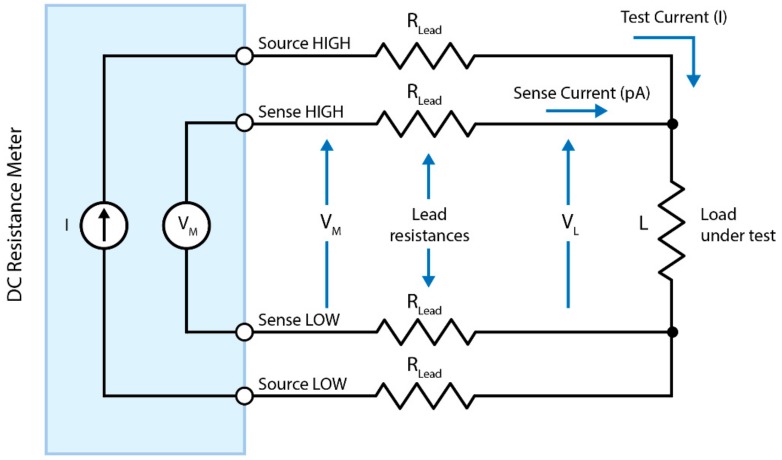
A four-point probe technique for measuring the resistivity of a sample under load.

A four-point probe, also known as a kelvin probe, as the name suggests uses four probes to overcome these problems. Figure 7 below shows the principle of operation of a four-point probe. In this configuration, the test current (*I*) is forced through the load via one set of source leads, while the voltage across the load (*V_L_*) is measured through a second set of sense leads. Although some small current (typically <100 pA) may flow through the sense leads, it is usually negligible and can be ignored for practical purposes. Therefore, the voltage measured by the voltmeter (*V_M_*) is essentially the same as the voltage across the load (*V_L_*). As a result, the resistance value can be determined more accurately using the test current *I* and Ohm’s law as *V_M_* = *V_L_*.

The measurement of electrical conductivity of metallic samples is quite straightforward provided the sample has contact pads for the probes and the test equipment is calibrated. For inks of a polymeric nature, such as semiconductors, it is a more complicated process, as the conductivity varies with temperature and humidity. In conducting polymers, the presence of conjugated double bonds along the backbone allows electrons to delocalize into a conduction band, forming an energy gap in the electronic spectrum [112]. In order to overcome the energy gap, charge-carrying dopant ions are introduced in the structure and consequently lead to conductivity through the semiconductor material [113]. The measurement of electrical conductivity on their surface is sensitive to humidity and temperature due to the presence of these ions [114].

As Equation (3) measures the volume resistivity, the cross-sectional area of the printed sample requires measurement. In the case of printed electronics, even the thickest printed films measure a few µm (10^−6^), and as such a profilometer is often required to obtain an accurate measurement of the thickness of the sample.

Table 2 below provides a brief overview of some of the most notable inkjet-printed inks, including copper and silver nanoparticle inks (CuNPs and AgNPs), as well as silver metal−organic decomposition (MOD) and silver reactive organometallic inks (ROM). The inks have been widely used, MOD inks being popular in 1988 via Teng and Vest et al. [115,116] with reliable results, depending on the sintering method chosen. Substrates varying from standard laboratory glass slides to Kapton polyimide (PI), polyethylene naphthalate (PEN), polyethylene terephthalate (PET) films, silicon wafers, and even paper with varying film thicknesses were printed onto and a form of post-treatment was applied to increase the conductivity of the inks.

The adhesion of the printed sample to the substrate is a key criterion for longevity, and is greatly influenced by the surface properties of the substrate, the wetting and penetration of the ink into the substrate, as well as the composition of the ink [130]. The sintering process is another important parameter which has a significant impact on the adhesion of the ink to the substrate. The solvent evaporation rate, the ink drying time, and the sintering temperature are parameters that are controlled through sintering. Uncontrolled sintering often leads to the formation of cracks due to excessive tension in the printed layers, leading to a poor surface adhesion or separation of layers.

**Figure 8:**
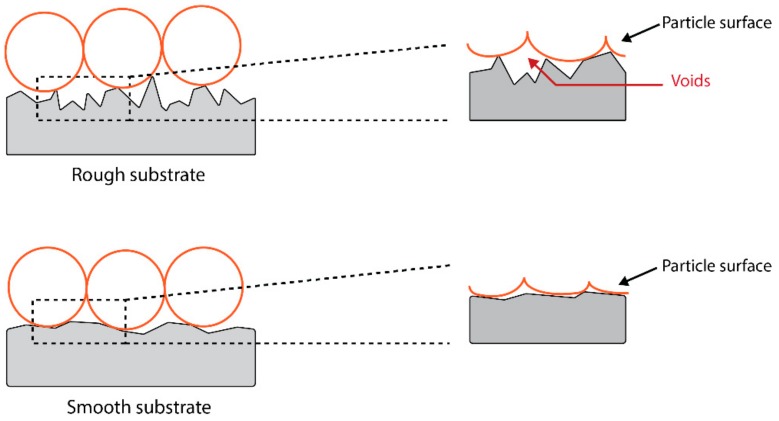
Comparison of the adhesion of particles on a rough surface and a smooth surface. The zoomed-in views show the formation of voids during the post–deposition processing, especially when the surface is rough.

In a metal-polymer interface, diffusion, absorption, and electrostatic forces can be considered negligible due to their relatively low significance as compared to mechanical interlocking and chemical bonding mechanisms [131]. In this scenario, mechanical interlocking describes the anchoring of the constituents of the ink (e.g., nanoparticles) to the substrate (e.g., glass), as shown in Figure 8 below. As a result, additional energy is required to separate the ink and the substrate. However, in some cases the increased surface roughness of the substrate can have the adverse effects of adhesive strength due to the failure of the constituents of the ink to penetrate the troughs, especially when the particles are larger than the troughs. This causes the formation of voids where moisture can be trapped, hence undermining the adhesive strength.

Chemical bonding is achieved by various mechanisms such as ionic, covalent, and metallic bonds as well as hydrogen bonds [132]. Identifying each chemical bond across a metal-polymer interface is impossible because of the complexity of the bonds and the inability to properly analyze the interface layer at such a nano scale. Thus, experimentally it is generally assumed that a set of chemical bonds contribute to the interfacial fracture. Intermolecular bonding forces between molecules, also known as Van der Waals forces, can be neglected predominantly due to the particular size being much less than 1 μm in diameter [133].

Capillary force is the force between solid and liquid due to the surface tension of the liquid. For the same particle size, the magnitude of the capillary force is known to be higher than that of the Van der Waals forces. However, during the nanoparticle sintering process, all solvents are assumed to be evaporated, and thus capillary force could be assumed as an insignificant factor in nanoparticle adhesion.

**Figure 9:**
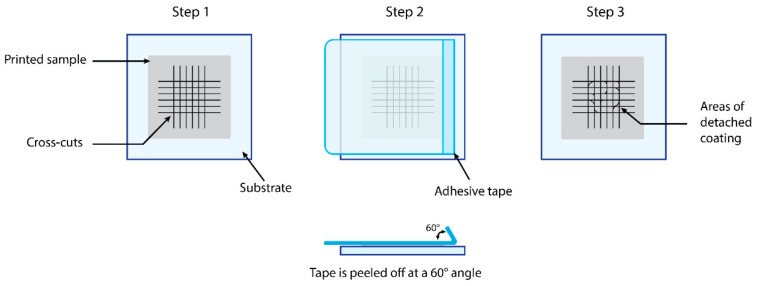
Illustration of the three-step process for visually assessing the cross-cut test.

There are two methods, both ISO-standardized and widely used in industry to quantify the adhesion of an ink to a substrate. The first method, known as the cross-cut test (ISO 2409) [134], is a means of assessing the resistance of a coating to separation from substrates. It is a multi-step process used in laboratories, shown in Figure 9 below, whereby a cutting tool is used to cut in a right-angle lattice pattern into the coating, penetrating through to the substrate. Six cuts should be placed in each direction of the lattice pattern, the spacing of which depends on the thickness of the coating and the type of substrate. For coatings of up to 120 μm thick, the cuts should be done at a spacing of 2 mm. After the cuts have been done, the sample is lightly brushed, and a piece of adhesive tape is placed over the lattice in the direction parallel to one set of cuts. Within 5 min of applying the tape, the tape is removed at an angle close to 60° at a steady rate of 0.5–1.0 s/cm. Finally, the sample is analyzed and cross-referenced to the table [134] in the ISO 2409 standard and a visual evaluation is reported.

**Figure 10:**
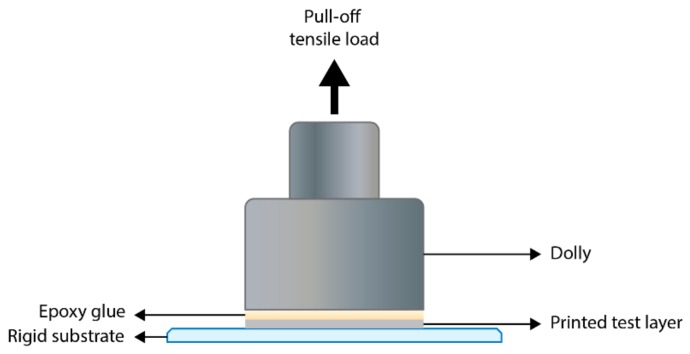
Schematic of the assembly for the pull-off test method.

The second method, known as the pull-off test (ISO 4624), is used to quantify the adhesion of a coating to a substrate through mechanical testing. In this method, an aluminum dolly is glued to the specimen and a vertical tensile load is applied perpendicular to the sample surface until failure, as shown in Figure 10 below. The force at which this occurs, and the type of failure obtained is recorded as a measure of the adhesion properties of the coating. There are several variables to this test method, including the choice of the epoxy glue and its composition, surface preparation, the design of the dolly, the curing conditions, as well as the consistency in the application of the vertical tensile load. Sridhar’s [131] work on the pull-off test method highlighted the importance of the selection of the glue in order to obtain significant results. The dolly–glue adhesion and the specimen–glue adhesion should be stronger than the cohesive strength of the printed layer, or the interfacial strength at the substrate. The vertical tensile load required to fracture the weakest interface is the outcome of the test and it can either be cohesive, adhesive, or cohesive-adhesive.

**Figure 11:**
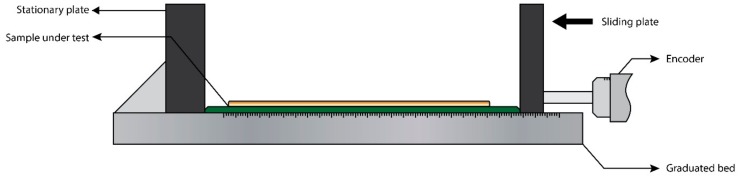
Illustration of a simple bending test for printed electronics. One plate is kept stationary while the other slides along the graduated bed, the distance of which is according to the test radius. The encoder is used to automate the process.

The adhesion and conductivity tests for printed electronics often end at this stage, provided the minimum requirements for performance are met. However, in the case of stretchable or flexible printed electronics, the final test necessitates a mechanical deformation in the form of a bending test. The design for the bending rig varies depending on the sample to be tested, but in essence it consists of a stage with two plates, a stationary and a sliding one, as shown in Figure 11. The sample is then pinned at the stationary plate end while the sliding plate is then moved in the direction of the stationary plate, causing a change in the bending radius. The sliding plate mechanism can be motorized to automate the process using an encoder, and at the same time perform a fatigue bending test to study the performance of the sample. During the deformation test, the electrical conductivity of the sample can be monitored and is then compared to the data prior to bending to assess the results after the deformation. The sample adhesion is also reassessed, as in many cases the deformation causes the delamination of the printed ink to the substrate [135,136].

## 10. Conclusions

Printed electronics is a diverse and growing field in which inkjet printing plays a prominent role, although it should be stressed that other printing techniques, such as gravure, are also employed due to their high throughput allowing for the rapid mass production of low-end electronic devices, such as sensors and RFID tags. However, regardless of the differences in the means of deposition, all of the techniques require a good understanding of the interactions between ink and substrate. This understanding is essential to ensure good print morphology. The means of converting a dried (or drying) deposited metallic ink into a conductive metal track is also common to all printing techniques, and all of the conversion methods (e.g., thermal, intense pulsed light and laser) have their advantages with the key driver being speed of conversion as well as electrical performance.

Returning to inkjet, one of the main printing parameters is dot spacing, which is the distance between the centers of two droplets; which can also be thought of as the degree of overlap. The smaller the dot spacing, the more ink per unit length/area, which has an effect on the end performance, particularly for conductive features. A small dot spacing will result in a more conductive track, as will increasing the number of deposited layers, but both approaches will result in an increase in production time, as well as longer post-printing processes due to the thicker features.

The form of the ink also has an influence. Most, if not nearly all, commercial inks are suspensions of nanoparticles, which upon deposition require the surfactant to be removed so that a conductive whole can be formed. Metal salt solution (metallo-organic decomposition) inks are a second form of ink that are simpler in terms of composition and give higher performances, but have some drawbacks, such as lower loadings and higher contact resistance.

Thermal processing has been typically employed in converting the as-printed deposit to the final conductive feature, but other techniques, such as intense pulsed light, are increasingly being used due to their higher throughput. The exact method of post-printing process influences the adhesion of the printed electronic device, with higher temperatures giving higher conductivities, but not necessarily better adhesion.

## Figures and Tables

**Table 1:** Comparison of available inkjet printing systems with respect to their performance and unique features.

System Type	Examples
Printheads for industrial applications	Xaar, Hitachi Ricoh, Konica Minolta, Kyocera
Laboratory & research systems	Microdrop, MicroFab, Fujifilm Dimatix

**Table 2:** A summary of some of the best experiments using high-performance inks and their sintering methods, with state-of-the-art results obtained. Abbreviations: reactive organometallic inks, ROM; metal−organic decomposition, MOD; intense pulsed light, IPL; near-infrared, NIR; poly(diallyldimethylammonium chloride), PDAC.

Author	Ink Type and Substrate	Sintering Method	Result (% Bulk Conductivity)
Niittynen et al. [117]	CuNPs on silicon wafer	808 nm continuous wave laser	23.4%
Niittynen et al. [118]	CuNPs on Kapton PI	808 nm continuous wave laser and IPL sintering	22%
Chan et al. [119]	CuNPs on PI film	Low pressure drying, NIR sintering and IPL exposure	21.3%
Kang et al. [120]	CuNPs on glass–epoxy flexible composite	200 °C in N_2_ gas atmosphere for 1 h	43.3%
Huang et al. [121]	Gold nanocrystals on plastic substrates	150 °C for 30 min	70%
Perelaer et al. [122]	AgNPs on PEN foil	Flash sintering and microwave sintering	40%
Perelaer et al. [123]	AgNPs and additives on boron silicate glass	Between 80 °C to 600 °C in an oven	Up to 56%
Niittynen et al. [124]	AgNPs on PI film	Photonic sintering	Up to 49%
Magdassi et al. [125]	AgNPs in PDAC solution printed on glass, PET and paper	Room temperature chemical conversion	20%
Black et al. [126]	Silver ROM ink on glass	120 °C in an oven	39.2%
Jahn et al. [127]	Silver MOD ink on glass and PET	250 °C in an oven	43%
Smith et al. [128]	Silver MOD ink on glass	150 °C on a hotplate for 5 min	53%
Valeton et al. [129]	Silver MOD ink on PET	Room temperature chemical reaction	10%
